# Walking into aging: real-world mobility patterns and digital benchmarks from the InCHIANTI Study

**DOI:** 10.1038/s41514-025-00245-w

**Published:** 2025-07-05

**Authors:** Jose Albites-Sanabria, Pierpaolo Palumbo, Stefania Bandinelli, Ilaria D’Ascanio, Sabato Mellone, Anisoara Paraschiv-Ionescu, Arne Küderle, Andrea Cereatti, Silvia Del Din, Felix Kluge, Eran Gazit, Carl-Philipp Jansen, Laura Delgado-Ortiz, Judith Garcia-Aymerich, Lynn Rochester, Jochen Klenk, Luigi Ferrucci, Clemens Becker, Lorenzo Chiari, Luca Palmerini

**Affiliations:** 1 Department of Electrical, Electronic, and Information Engineering “Guglielmo Marconi”, University of Bologna, Bologna, Italy; 2 Azienda Sanitaria Toscana Centro, Firenze, Piero Palagi Hospital, Firenze, Italy; 3 Health Sciences and Technologies-Interdepartmental Center for Industrial Research, University of Bologna, Bologna, Italy; 4 Laboratory of Movement Analysis and Measurement, Ecole Polytechnique Federale de Lausanne, Lausanne, Switzerland; 5 Machine Learning and Data Analytics Lab, Department of Artificial Intelligence in Biomedical Engineering, Friedrich-Alexander-Universität Erlangen-Nürnberg, Erlangen, Germany; 6 Department of Electronics and Telecommunications, Politecnico di Torino, Turin, Italy; 7 National Institute for Health and Care Research (NIHR) Newcastle Biomedical Research Centre (BRC), Newcastle University and the Newcastle Upon Tyne Hospitals NHS Foundation Trust, Newcastle Upon Tyne, UK; 8 Translational and Clinical Research Institute, Newcastle University, Newcastle upon Tyne, UK; 9 Biomedical Research, Novartis Pharma AG, Basel, Switzerland; 10 Center for the Study of Movement, Cognition and Mobility, Neurological Institute, Tel Aviv Sourasky Medical Center, Tel Aviv, Israel; 11 Department of Clinical Gerontology, Robert Bosch Hospital, Stuttgart, Germany; 12 Geriatric Center, Heidelberg University Medical Center, Heidelberg, Germany; 13 ISGlobal, Barcelona, Spain; 14 Universitat Pompeu Fabra (UPF), Barcelona, Spain; 15 CIBER Epidemiología y Salud Pública (CIBERESP), Barcelona, Spain; 16 Department of Sport, Exercise and Health, University of Basel, Basel, Switzerland; 17 Institute of Epidemiology and Medical Biometry, Ulm University, Ulm, Germany; 18 IB University of Health and Social Sciences, Stuttgart, Germany; 19 Translational Gerontology Branch, National Institute on Aging, National Institutes of Health, Bethesda, MD USA

**Keywords:** Biomarkers, Health care, Medical research, Risk factors

## Abstract

Mobility is a cornerstone of health and quality of life, particularly in older adults. Digital mobility outcomes (DMOs) from real-world walking data offer crucial insights into the functional status and early markers of mobility decline. This study provides reference values for walking activity, pace, rhythm, and gait bout-to-bout variability in community-dwelling older adults and evaluates the effects of age, sex, height, and weight on these parameters. Using data from 200 older adults (aged 65–94 years) from the InCHIANTI Study and applying the Mobilise-D computational pipeline, we analyzed real-world walking over a week. Significant differences by sex and age were found, with males showing higher walking activity in younger age groups (65–74 and 75–84 years) but not in the oldest group (85–94 years). Additionally, we observed non-linear trends in mobility metrics with age, indicating an accelerated reduction in mobility at certain age ranges. These findings underscore the importance of monitoring real-world walking data to pinpoint critical periods of mobility decline and guide targeted interventions. This work offers valuable benchmarks for clinical assessments and future research.

## Introduction

In aging societies, maintaining independence and autonomy is increasingly recognized as a paramount component of healthy living. Among the factors that influence independent living, mobility stands out as a critical element^1^. As individuals age, physical impairments related to chronic conditions like arthritis, cardiovascular diseases, and neurological disorders become more common, often leading to restricted mobility^2^. This gradual decline not only impacts an individual’s capacity to perform everyday tasks but also diminishes their quality of life. This makes mobility a central focus of healthcare interventions and aging research.

The global rise in the prevalence of mobility limitations has been identified as a significant challenge by public health authorities. According to the World Health Organization (WHO), millions of individuals, particularly older adults, experience some form of mobility disability^3^. These limitations result from a range of impairments affecting multiple systems, from musculoskeletal to respiratory and neurological, which progressively compromise an individual’s ability to move independently. This growing trend is expected to accelerate as populations age and chronic diseases become more prevalent, imposing economic and social burdens on individuals and societies.

Traditionally, healthcare professionals have relied on clinical tests to evaluate mobility, such as the Timed Up and Go (TUG) test, Short Physical Performance Battery (SPPB), gait speed assessments, and the 6-Minute Walk Test (6MWT)^1^. These structured walking assessments conducted in controlled environments provide standardized measures of functional mobility. While these methods provide helpful insights into an individual’s physical capacity, they fall short in assessing real-world mobility performance and lack ecological validity^4,5^. The challenge lies in understanding how individuals move and function in their day-to-day lives outside of clinical settings. Relying solely on episodic assessments provides an incomplete picture of mobility, overlooking important fluctuations in gait patterns, activity levels, and the extreme variability in the environment in which movement occurs. As a result, there is a pressing need for innovative solutions that offer continuous, real-life assessments of mobility.

Recent advancements in digital health technologies have transformed how mobility can be measured^6^. Wearable devices enable the continuous capture of data on physical activity, providing a comprehensive overview of how individuals move within their daily environments. These sensors can track various movements and deliver precise, objective metrics that accurately reflect an individual’s mobility^7–10^. From this data, digital mobility outcomes (DMOs) can be extracted, providing healthcare providers with a robust set of indicators to monitor mobility over time. However, while the potential usefulness is significant, developing accurate algorithms to derive these DMOs and ensuring that the findings are clinically valid across different settings and populations remain crucial challenges for the field.

The Mobilise-D consortium^11^ has made significant progress in digital mobility assessment by developing and validating algorithms to extract digital mobility outcomes (DMOs) from real-world walking data. These advancements have addressed many technical and clinical challenges, ensuring the accuracy, reliability, and ecological validity of DMOs^12–15^. Through rigorous validation processes, the Mobilise-D consortium has demonstrated the generalizability of these algorithms across various cohorts, including healthy older adults and patients with Parkinson’s disease, multiple sclerosis, chronic obstructive pulmonary disease, and proximal femoral fracture^13,16^. The Mobilise-D computational pipeline, which consists of validated algorithms to derive DMOs, is currently used in their Clinical Validation Study to provide longitudinal data on the use of digital mobility outcomes to identify, stratify, and monitor disability^17^.

In this state-of-the-art study, we applied the Mobilise-D computational pipeline (using a local implementation for usage on external datasets) on data collected in community-dwelling older subjects from a population-based study of aging based in the Chianti region of Tuscany, Italy (InCHIANTI)^18^. Our study aims to provide reference values for real-world walking patterns in community-dwelling older adults. For this purpose, we (1) identify real-world walking events to extract 24 distinct DMOs and (2) examine the influence of age, sex, height, and weight on these metrics, providing population reference values and their characteristics.

## Results

Twenty-four DMOs were derived from real-world walking data of 200 community-dwelling older adults (Table 1, Supplementary Figs. 1 and 2). Participants had an average of 6.3 (sd = 1.5) valid wear days during the week of monitoring. Among them, 188 participants had more than 5 valid days, and only 12 participants had 3–4 valid days.

**Table 1:** Digital mobility outcomes (DMOs) in the InCHIANTI study. Refer to Table 6 for DMO definitions

	Digital mobility outcome	Mean (sd)	Median (P25–P75)
1	Walking duration [h/day]	1.6 (0.7)	1.5 (1.1–2.0)
2	Number of steps [steps/day]	8524.8 (3659.1)	8261.5 (5963.8–10913.2)
3	Number of WBs [WB/day]	378 (139)	379 (281–475)
4	Number of WBs >10 s [WB/day]	157 (65)	155 (112–200)
5	Number of WBs >30 s [WB/day]	30 (17)	26 (17–40)
6	Number of WBs >60 s [WB/day]	10 (7)	8 (5–13)
7	WBs duration [s]	8.6 (0.9)	8.5 (8.0–9.1)
8	P90 WBs duration [s]*	27.6 (9.8)	25.5 (21.7–30.3)
9	WBs duration bout to bout variability [–]	1.5 (0.6)	1.4 (1.1–1.8)
10	Walking speed in shorter (10–30 s) WBs [m/s]	0.64 (0.07)	0.64 (0.60–0.68)
11	Walking speed in longer (>30 s) WBs [m/s]	0.79 (0.11)	0.79 (0.73–0.87)
12	P90 walking speed in WBs >10 s [m/s]	0.86 (0.12)	0.86 (0.78–0.95)
13	P90 walking speed in longer (>30 s) WBs [m/s]	0.96 (0.16)	0.97 (0.86–1.08)
14	Stride length in shorter (10–30 s) WBs [cm]	87.80 (8.63)	88.01 (82.02–93.66)
15	Stride length in longer (>30 s) WBs [cm]	103.29 (11.83)	103.69 (96.18–111.02)
16	Cadence in all WBs [steps/min]	84.83 (4.65)	84.67 (81.42–87.79)
17	Cadence in longer (>30 s) WBs [steps/min]	91.39 (6.48)	91.68 (86.57–96.21)
18	P90 cadence in longer (>30 s) WBs [steps/min]	101.30 (8.04)	102.04 (95.79–107.06)
19	Stride duration in all WBs [s]	1.29 (0.06)	1.29 (1.25–1.33)
20	Stride duration in longer (>30 s) WBs [s]	1.26 (0.08)	1.25 (1.20–1.30)
21	Walking speed bout to bout variability in longer (>30 s) WBs [–]	0.18 (0.05)	0.18 (0.15–0.21)
22	Stride length bout to bout variability in longer (>30 s) WBs [–]	0.13 (0.03)	0.12 (0.11–0.15)
23	Cadence bout to bout variability [–]	0.12 (0.01)	0.12 (0.11–0.13)
24	Stride duration bout to bout variability [–]	0.15 (0.02)	0.15 (0.14–0.16)

^*^Non normally distributed; the 90th percentile (P90) is also referred to as ‘Maximum’^34,38^.

Subgroup analyses (Table 2) by sex and age revealed differences in gait quality metrics that varied across age groups (65–74, 75–84, and 85–94 years). In the 65–74 years age group, males demonstrated significantly longer walking duration (2.4 h/day vs. 1.7 h/day, *p* = 0.002), higher daily step counts (12,968 vs. 9660 steps/day, *p* = 0.004), and more walking bouts (WBs) longer than 30 s (58/day vs. 30/day, *p* < 0.001) and 60 s (18/day vs. 9/day, *p* < 0.001), as well as a longer WBs duration (9.52 s vs. 8.39 s, *p* < 0.001). Males in this group also exhibited longer stride lengths in both shorter WBs (95.6 cm vs. 89.3 cm, *p* = 0.004) and longer WBs (113.9 cm vs. 103.5 cm, *p* < 0.001). Similar patterns were observed in the 75–84 years age group, where males had a higher number of WBs > 30 s (31/day vs. 24/day, *p* = 0.032), WBs > 60 s (11/day vs. 8/day, *p* = 0.019), and a longer WBs duration (8.8 s vs. 8.2 s, *p* < 0.001), as well as longer stride lengths in shorter WBs (91.0 cm vs. 85.0 cm, *p* < 0.001) and longer WBs (110.0 cm vs. 98.2 cm, *p* < 0.001). In the 85–94 years age group, however, sex differences were less pronounced. While males had longer stride lengths in shorter WBs (86.3 cm vs. 78.5 cm, *p* = 0.049) and females showed higher cadence in all WBs (87.45 steps/min vs. 82.52 steps/min, *p* = 0.017), other metrics such as walking duration, step counts, and WBs characteristics no longer differed significantly between sexes.

**Table 2:** DMOs differences by sex and age, adjusted by height and BMI. Significant *p*-values (*p* < 0.05) are shown in bold

	65–74 (*n* = 45)			75–84 (*n* = 109)			85–94 (*n* = 46)		
DMO	Female (*n* = 28)	Male (*n* = 17)	*p*-Value	Female (*n* = 53)	Male (*n* = 56)	*p*-Value	Female (*n* = 17)	Male (*n* = 29)	*p*-Value
Walking duration [h/day]	1.7 (0.5)	2.4 (0.8)	**0.002**	1.5 (0.6)	1.5 (0.6)	0.895	1.2 (0.5)	1.4 (0.6)	0.675
Number of steps [steps/day]	9660 (2773)	12968 (4303)	**0.004**	8427 (3574)	7979 (3267)	0.503	6594 (2585)	7189 (3265)	0.724
Number of WBs [WB/day]	448 (115)	486 (135)	0.311	396 (136)	339 (129)	0.032	332 (124)	318 (134)	0.712
Number of WBs >10 s [WB/day]	182 (55)	231 (74)	**0.019**	155 (58)	144 (60)	0.411	124 (51)	136 (61)	0.724
Number of WBs >30 s [WB/day]	30 (11)	58 (21)	**<0.001**	24 (13)	31 (16)	**0.03**	18 (10)	27 (15)	0.105
Number of WBs >60 s [WB/day]	9 (4)	18 (7)	**<0.001**	8 (6)	11 (7)	**0.01**	5 (4)	9 (6)	0.123
WBs duration [s]	8.4 (0.6)	9.5 (0.8)	**<0.001**	8.2 (0.8)	8.8 (0.9)	**<0.001**	8.1 (0.9)	8.7 (1.1)	0.121
P90 WBs duration [s]	24.8 (3.0)	33.8 (4.6)	**<0.001**	23.5 (4.7)	31.1 (11.1)	**<0.001**	22.7 (4.0)	30.6 (16.0)	0.051
WBs duration bout to bout variability [–]	1.5 (0.5)	1.7 (0.6)	0.184	1.5 (0.6)	1.6 (0.6)	0.441	1.3 (0.6)	1.4 (0.5)	0.684
Walking speed in shorter (10–30 s) WBs [m/s]	0.68 (0.04)	0.68 (0.05)	0.716	0.63 (0.05)	0.64 (0.06)	0.789	0.59 (0.07)	0.61 (0.08)	0.724
Walking speed in longer (>30 s) WBs [m/s]	0.84 (0.07)	0.89 (0.09)	0.052	0.76 (0.06)	0.83 (0.10)	**<0.001**	0.71 (0.12)	0.73 (0.14)	0.746
P90 walking speed in WBs >10 s [m/s]	0.91 (0.07)	1.02 (0.09)	**<0.001**	0.82 (0.07)	0.90 (0.11)	**<0.001**	0.75 (0.11)	0.80 (0.15)	0.46
P90 walking speed in longer (>30 s) WBs [m/s]	1.0 (0.1)	1.1 (0.1)	**0.004**	0.9 (0.1)	1.0 (0.2)	**<0.001**	0.8 (0.2)	0.9 (0.2)	0.684
Stride length in shorter (10–30 s) WBs [cm]	89.3 (6.0)	95.6 (6.8)	**0.004**	85.0 (6.2)	91.0 (8.4)	**<0.001**	78.5 (7.0)	86.3 (9.9)	**0.049**
Stride length in longer (>30 s) WBs [cm]	103.5 (7.8)	113.9 (8.9)	**<0.001**	98.2 (8.1)	110.0 (10.3)	**<0.001**	92.2 (11.3)	99.8 (14.0)	0.15
Cadence in all WBs [steps/min]	87.9 (3.3)	83.3 (2.8)	**<0.001**	86.7 (4.6)	82.3 (3.5)	**<0.001**	87.4 (4.7)	82.5 (4.7)	**0.017**
Cadence in longer (>30 s) WBs [steps/min]	96.5 (4.9)	92.1 (4.9)	**0.012**	92.9 (5.8)	89.2 (5.7)	**<0.001**	92.0 (7.0)	87.2 (7.1)	0.051
P90 cadence in longer (>30 s) WBs [steps/min]	107.7 (6.2)	103.8 (5.9)	0.052	102.8 (7.0)	98.8 (7.4)	**0.003**	100.7 (8.5)	96.1 (8.7)	0.121
Stride duration in all WBs [s]	1.26 (0.04)	1.29 (0.04)	0.061	1.28 (0.06)	1.32 (0.07)	**0.013**	1.26 (0.07)	1.32 (0.06)	**0.031**
Stride duration in longer (>30 s) WBs [s]	1.21 (0.06)	1.24 (0.05)	0.115	1.25 (0.08)	1.28 (0.09)	0.098	1.24 (0.08)	1.30 (0.08)	**0.049**
Walking speed bout to bout variability in longer (>30 s) WBs [–]	0.18 (0.03)	0.23 (0.03)	**<0.001**	0.17 (0.05)	0.19 (0.05)	**0.049**	0.15 (0.05)	0.16 (0.04)	0.46
Stride length bout to bout variability in longer (>30 s) WBs [–]	0.13 (0.02)	0.15 (0.03)	**0.016**	0.12 (0.03)	0.13 (0.04)	0.098	0.12 (0.04)	0.12 (0.03)	0.712
Cadence bout to bout variability [–]	0.13 (0.01)	0.13 (0.01)	0.94	0.12 (0.01)	0.12 (0.01)	0.277	0.12 (0.02)	0.11 (0.01)	0.602
Stride duration bout to bout variability [–]	0.15 (0.01)	0.16 (0.02)	**0.008**	0.14 (0.02)	0.15 (0.02)	**<0.001**	0.15 (0.02)	0.15 (0.02)	0.756

**Fig. 1:**
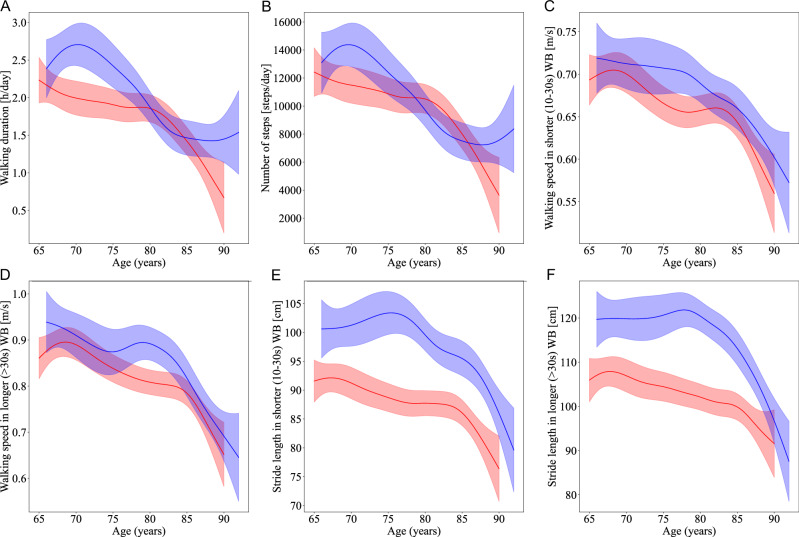
Age-related changes in real-world digital mobility outcomes. **A**–**F** Generalized additive model (GAM) regression curves show how digital mobility outcomes (DMOs) vary with age in females (red) and males (blue). Shaded areas indicate 95% confidence intervals. Explained variance (*R*^2^) and statistical significance (*p < 0.05, **p < 0.01, ***p < 0.001) are reported for each DMO. **A** Walking duration (hours per day) shows non-linear decreases with advancing age, with *R*^2^ = 41.1% ** (females) and 45.2% *** (males). **B** Number of steps per day decreases with age, with *R*^2^ = 42.6% ** (females) and 44.9% *** (males). **C** Walking speed during short walking bouts declines steadily across age groups, with *R*^2^ = 43.6% * (females) and 39.3% (males). **D** Walking speed in long walking bouts follows a similar trend, with *R*^2^ = 47.4% ** (females) and 46.2% *** (males). **E** Stride length declines with age, with *R*^2^ = 45.6% (females) and 44.1% ** (males). **F** Cadence also exhibits reductions with aging, with *R*^2^ = 41.1% (females) and 52.3% *** (males).

Supplementary Table 2 presents the results of the generalized additive models (GAMs) fitted to assess the non-linear relationship between each DMO and age, stratified by sex and adjusted for height and weight. Figure 1 displays the non-linear age trends and 95% confidence intervals for selected DMOs, illustrating how specific mobility parameters, such as walking duration, number of steps, walking speed, and stride length, change with age.

**Fig. 2:**
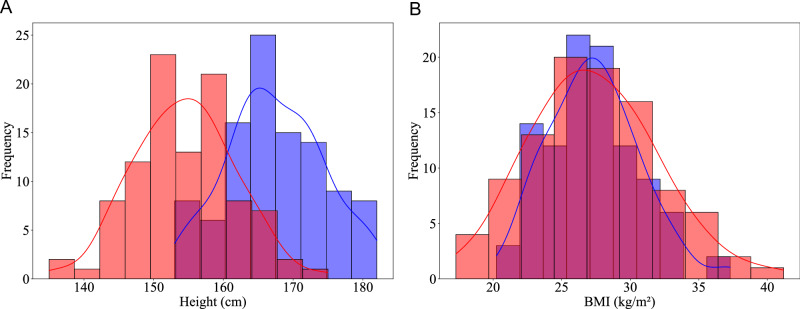
Distribution of height and body mass index (BMI) by sex. **A**, **B** Distributions of height and body mass index (BMI) are plotted separately for males (blue) and females (red). Vertical bars represent histogram bins; overlaid curves indicate kernel density estimates derived from each distribution. **A** Height distributions (cm) for females (red, *n* = 98) and males (blue, *n* = 102) **B** BMI distributions (kg/m^2^) for females (red, *n* = 98) and males (blue, *n* = 102).

The distribution of height and BMI among males and females is presented in Fig. 2. Male participants had a median height of 167 (163–172) cm, whereas female participants had a median height of 155 (150–159) cm, with a significant difference between sexes (*p* < 0.001). In contrast, BMI showed overlapping distributions between males and females, with median values of 27.0 and 27.2 kg/m^2^, respectively, and no significant difference (*p* = 0.218).

Table 3 presents the analysis of covariance (ANCOVA) to assess differences in DMOs between height categories (‘short’ vs ‘tall’). Among female participants, several DMOs showed significant differences between height groups. Walking speed was significantly greater in taller females compared to shorter females, both in shorter (0.66 m/s vs. 0.62 m/s, *p* = 0.0004) and longer duration walking bouts (0.80 m/s vs. 0.75 m/s, *p* = 0.002). P90 walking speed in bouts longer than 10 s was also greater in taller females (0.87 m/s vs. 0.80 m/s, *p* = 0.0005). For males, however, none of the DMOs showed significant differences between height groups.

**Table 3:** DMOs grouped by height and sex differences. Significant *p*-values (*p* < 0.05) are shown in bold

	Female				Male		
DMOs	Short (*n* = 48)	Tall (*n* = 50)	*p*-Value	Short (*n* = 50)		Tall (*n* = 52)	*p*-Value
Walking duration [h/day]	1.6 (0.6)	1.4 (0.5)	0.340	1.6 (0.7)		1.6 (0.7)	0.908
Number of steps [steps/day]	8962 (3638)	7981 (2972)	0.301	8690 (3973)		8397 (3974)	0.908
Number of WBs [WB/day]	414 (132)	387 (134)	0.481	370 (154)		341 (128)	0.887
Number of WBs >10 s [WB/day]	162 (61)	153 (57)	0.569	158 (74)		154 (67)	0.908
Number of WBs >30 s [WB/day]	26 (14)	23 (12)	0.386	34 (20)		34 (20)	0.999
Number of WBs >60 s [WB/day]	8 (5)	7 (4)	0.481	12 (8)		11 (7)	0.908
WBs duration [s]	8.2 (0.8)	8.3 (0.7)	0.673	8.7 (1.0)		9.1 (0.8)	0.321
P90 WBs duration [s]	3.2 (0.2)	3.1 (0.2)	0.569	3.4 (0.3)		3.4 (0.3)	0.943
WBs duration bout to bout variability [–]	0.4 (0.4)	0.3 (0.3)	0.275	0.4 (0.3)		0.4 (0.3)	0.908
Walking speed in shorter (10–30 s) WBs [m/s]	0.62 (0.06)	0.66 (0.05)	**<0.001**	0.62 (0.07)		0.65 (0.07)	0.472
Walking speed in longer (>30 s) WBs [m/s]	0.75 (0.09)	0.80 (0.08)	**<0.001**	0.80 (0.12)		0.81 (0.13)	0.908
P90 walking speed in WBs >10 s [m/s]	0.80 (0.10)	0.87 (0.08)	**<0.001**	0.88 (0.13)		0.90 (0.15)	0.908
P90 walking speed in longer (>30 s) WBs [m/s]	0.9 (0.1)	1.0 (0.1)	**0.011**	1.0 (0.2)		1.0 (0.2)	0.908
Stride length in shorter (10–30 s) WBs [cm]	81.9 (7.1)	88.1 (6.0)	**<0.001**	88.5 (8.3)		92.2 (9.6)	0.321
Stride length in longer (>30 s) WBs [cm]	95.3 (9.4)	101.9 (8.1)	**<0.001**	106.0 (11.5)		109.2 (13.1)	0.716
Cadence in all WBs [steps/min]	87.6 (4.6)	86.9 (4.0)	0.569	82.6 (3.8)		82.3 (3.6)	0.908
Cadence in longer (>30 s) WBs [steps/min]	93.7 (6.3)	93.8 (5.8)	0.951	89.7 (6.3)		88.3 (5.7)	0.875
P90 cadence in longer (>30 s) WBs [steps/min]	103.9 (8.1)	103.7 (6.8)	0.936	100.0 (7.8)		97.5 (7.7)	0.472
Stride duration in all WBs [s]	1.27 (0.05)	1.27 (0.06)	0.936	1.31 (0.06)		1.32 (0.07)	0.955
Stride duration in longer (>30 s) WBs [s]	1.24 (0.07)	1.23 (0.08)	0.673	1.27 (0.08)		1.29 (0.09)	0.908
Walking speed bout to bout variability in longer (>30 s) WBs [–]	0.16 (0.04)	0.17 (0.05)	0.468	0.19 (0.05)		0.18 (0.05)	0.908
Stride length bout to bout variability in longer (>30 s) WBs [–]	0.12 (0.02)	0.13 (0.04)	0.548	0.13 (0.04)		0.13 (0.03)	0.908
Cadence bout to bout variability [–]	0.12 (0.01)	0.12 (0.01)	0.936	0.12 (0.01)		0.12 (0.01)	0.908
Stride duration bout to bout variability [–]	0.14 (0.02)	0.15 (0.02)	0.468	0.15 (0.01)		0.15 (0.02)	0.955

**Fig. 3:**
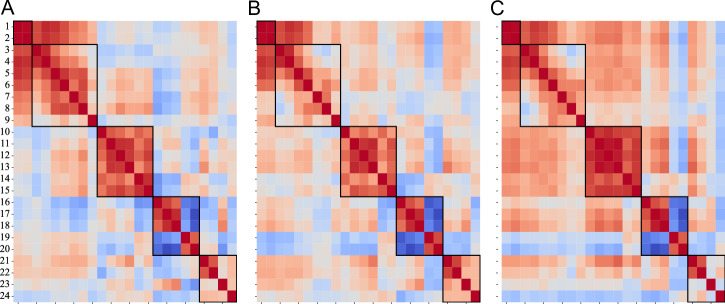
Age-specific correlation networks of digital mobility outcomes. **A**–**C** Correlation matrices are displayed as networks for three age groups: 65–74, 75–84, and 85–94 years. Cell color indicates strength and direction of correlation (blue for negative, red for positive). Marked edges are outlined by domain classification as defined in Table 6 (Amount, Pattern, Pace, Rhythm, and Variability). **A** In the 65–74 group, strong correlations correspond to each of five domains of mobility. Inter domain correlations are seen in the first two domains (amount and pattern). **B** The 75–84 network retains clustered connectivity but with slightly reduced overall correlations. **C** In the 85–94 group, connectivity patterns show an increase in inter-domain correlations indicating a greater overlap in mobility patterns.

Figure 3 displays the correlation matrices for the age groups 65–74, 75–84, and 85–94 years, the marked edges represent the five domains outlined in Table 6. As age increases, there is a notable rise in inter-domain correlations (up to 0.7), indicating a greater overlap in mobility characteristics. Consequently, in the oldest group, the distinctions between the marked domains become less pronounced.

Further investigation into the heatmaps analysis was conducted through factor analysis, which confirmed five distinct factors, as shown in Table 4. The five factors cumulatively explained 80% of the total variance in DMOs. Factors 1 and 2 revealed substantial overlap (the one with the strongest loading was kept). This overlap is further confirmed by the strong correlations of the first two domains in the heatmap, prompting the consolidation of these factors into a single suggested label termed “Walking Activity” (“Amount” and “Pattern”).

**Table 4:** Factor analysis of the 24 DMOs

Factor	DMOs	Explained variance	Labels
1	[‘Walking duration’, ‘Number of steps’, ‘Number of WBs’, ‘Number of WBs > 10 s’, ‘Number of WBs > 30 s’]	0.18	Walking Activity (amount + pattern)
2	[‘Number of WBs > 60 s’, ‘WBs duration ‘, ‘P90 WBs duration’, ‘ WBs duration bout to bout variability’]	0.12	
3	[‘Walking speed in shorter (10–30 s) WBs’, ‘Walking speed in longer (>30 s) WBs ‘, ‘P90 walking speed in WBs > 10 s’, ‘P90 walking speed in longer WBs ‘, ‘Stride length in shorter (10–30 s) WBs ‘, ‘Stride length in longer (>30 s) WBs ‘]	0.21	Pace
4	[‘Cadence in all WBs’, ‘Cadence in longer (>30 s) WBs ‘, ‘P90 cadence in longer WBs ‘, ‘Stride duration in all WBs’, ‘Stride duration in longer (>30 s) WBs ‘]	0.20	Rhythm
5	[‘Walking speed bout to bout variability in longer (>30 s) WBs’, ‘Stride length bout to bout variability in longer (>30 s) WBs’, ‘Cadence bout to bout variability’, ‘Stride duration bout to bout variability’]	0.09	Variability

**Fig. 4:**
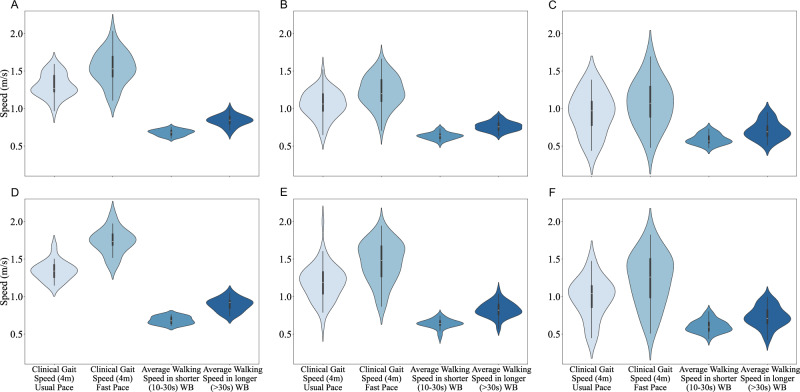
Clinical and real-world gait speed distributions by age and sex. **A**–**F** Box and violin plots compare gait speed across clinical (usual and fast pace) and real-world settings (short and long walking bouts) across six groups. Violin plots display the full distribution, with boxplots marking median and interquartile range. **A** Female participants aged 65–74 years (*n* = 28). **B** Female participants aged 75–84 years (*n* = 53). **C** Female participants aged 85–94 years (*n* = 17). **D** Male participants aged 65–74 years (*n* = 17). **E** Male participants aged 75–84 years (*n* = 56). **F** Male participants aged 85–94 years (*n* = 29).

In Fig. 4, we report a comparison between supervised clinical gait speed (from a 4-meter walking test) and unsupervised real-world walking speed (from walking speed in shorter and walking bouts) by age and sex groups. Comparison with P90 (‘Maximum’) walking speed in shorter and longer walking bouts is reported in Supplementary Fig. 3.

## Discussion

This study characterizes real-world walking in 200 community-dwelling older adults from the InCHIANTI study. DMOs were grouped into walking activity, pattern, pace, rhythm, and bout-to-bout variability through data-driven approaches. The baseline values for the 24 DMOs in our study population offer an overview of the typical mobility patterns in real-world walking for community-dwelling older adults aged 65 and above. The mean walking duration of the population was 1.6 h per day with an average number of 8525 steps per day, which aligns with recommended physical activity levels for older adults^19–21^. Walking speed in shorter walking bouts of 10–30 s was slower (0.64 m/s) than in longer walking bouts of >30 s (0.79 m/s). The maximum walking speed in longer walking bouts (expressed as the 90th percentile) was 0.96 m/s, which is within the range observed in real-world settings^15^. These values, however, are lower than average speeds reported in controlled clinical studies of older adults^22,23^, and they are below the average clinical gait speed during the 4 m walking test (1.1 m/s, usual pace) in this study. This discrepancy between laboratory and real-world DMOs could be attributed to the more challenging and variable environmental conditions encountered in real-world, unsupervised settings. In contrast, in controlled and supervised environments, individuals may try harder due to the presence of supervision and heightened awareness of being assessed (Hawthorne effect)^4,24^. The difference in walking speeds between shorter and longer walking bouts is consistent with studies showing trends toward higher speeds measured over longer, uninterrupted walking bouts^25^.

The subgroup analysis by sex revealed interesting differences. In the 65-74 years age group, males exhibited significantly higher walking duration (2.37 h/day vs. 1.72 h/day, *p* = 0.004) and step counts (12,967 vs. 9659 steps/day, *p* = 0.006) compared to females, suggesting greater mobility and physical activity in males during earlier stages of aging. However, as age increased, these differences diminished, becoming statistically insignificant for the 75–84 and 85–94 years groups. Additionally, males consistently had higher walking speeds in longer walking bouts (>30 s) compared to females in the 65–74 and 75–84 years age groups, which could be due to a variety of factors such as differing levels of physical activity, muscle strength, or overall health.^26,27^. This difference became again insignificant in the oldest group. These findings are consistent with a previous meta-analysis showing a decline in walking speeds in females as they age^28^. This convergence of DMOs in the oldest age group likely reflects the overall impact of physiological aging, where both males and females experience similar declines in mobility due to common factors such as frailty, chronic diseases, and reduced muscle strength. As these age-related factors become more dominant, they may overshadow the differences observed in younger age groups, leading to more similar gait characteristics between sexes.

Further analysis of height-based subgroups revealed some trends when participants were categorized as “short” or “tall” based on the median height for each sex group. For females, taller participants demonstrated significantly higher walking speeds in both shorter (10–30 s) and longer (>30 s) walking bouts, as well as greater P90 (‘Maximum’) walking speed within longer bouts. This trend aligns with previous research indicating that height contributes to gait dynamics, with taller individuals exhibiting longer strides and faster walking speeds due to biomechanical advantages, such as longer limbs and increased stride length^29^. Interestingly, these differences were not observed in males, suggesting that the observed height ranges in males in this population had no significant effect on the observed mobility patterns. Our findings here confirm and emphasize the importance of considering both height and sex when interpreting mobility outcomes in this population.

Correlation analyses revealed distinct inter-domain correlations of mobility metrics in younger adults (65–74 years), which became progressively less distinct with advancing age. This may reflect an increasing interdependence of mobility functions due to a general decline in neuromuscular function and adaptability^30,31^. In younger adults, specific DMO metrics—such as walking duration, number of steps, and number of WBs—were more independently pronounced, reflecting versatile and targeted mobility patterns. However, as mobility becomes limited in older adults, these metrics likely become less differentiated due to constraints on physical activity, consistent with previous studies suggesting a convergence of gait parameters with advanced age as a compensatory response to musculoskeletal decline^32^.

Factor analysis further supported the clustering observed in the heatmaps by identifying five distinct factors that explained 80% of the variance in DMOs. Among these factors, two (relating to “Amount” and “Pattern” of WBs) showed considerable overlap, prompting the suggestion of a unified “Walking Activity” category. High levels of activity (i.e., duration and frequency of walking bouts) and organized patterns of movement form a primary dimension of mobility in older adults, corroborating the “activity volume” hypothesis in gerontology, which posits that both the quantity and structure of movement are essential for maintaining function in aging populations^33^. The other factors—Pace, Rhythm, and Variability—align with established gait constructs, where each captures distinct yet interrelated aspects of movement. For example, Pace reflects speed and stride length, while Rhythm encompasses cadence and stride duration, both of which are closely tied to coordinated control and balance. Our findings are consistent with the aggregation proposed by the Mobilise-D consortium^34^.

The use of GAMs provided further insights into the complex, non-linear relationships between age and DMOs. GAM analyses revealed significant, non-linear trends in several mobility metrics, such as walking speed and stride length, with accelerated declines in specific age ranges. These findings suggest that the system remains relatively stable until a critical tipping point is reached. Beyond this threshold, functional limitations such as reduced outdoor activity or the transition to aged care result in a gradual, stepwise decline in mobility. Previous longitudinal studies on aging have observed similar non-linear trajectories, where individuals experience stable mobility for several years, followed by abrupt declines often associated with critical health events, such as the onset of chronic conditions or significant musculoskeletal deterioration^35,36^. In line with this, our models demonstrated higher explained deviance (pseudo-*R*^2^) for speed- and stride-related metrics in males, indicating a stronger model fit and suggesting that age trends in these metrics may be more pronounced in males. This sex-based difference could be due to variations in physical activity patterns, muscle composition, and health profiles between older males and females^26^. GAMs also revealed non-significant age trends in variability measures, which may reflect compensatory adaptations in gait stability that preserve variability metrics despite declining speed or stride length. This finding aligns with research indicating that variability in gait parameters may not decline linearly and could represent an independent construct linked to adaptive resilience in older adults^37^.

These findings underscore the complexity of mobility decline in older adults, highlighting distinct age and sex-specific patterns that offer important implications for mobility assessment and targeted interventions in geriatric care. The integration of height, BMI, and sex as covariates in these analyses emphasizes the need to account for these individual differences when assessing mobility in clinical and research settings. However, this study has several limitations that future research should address. Future studies should include more diverse populations, such as those with chronic conditions, lower socioeconomic backgrounds, and from varied environments and countries, to enhance the generalizability of findings. Notably, the InCHIANTI population in this study was relatively fit and healthy, which may limit the generalizability of the findings to other populations with varying health statuses. Additionally, while we focused on 24 digital mobility outcomes (DMOs), future studies should explore a broader range of gait measures, including irregular walking patterns, activity transitions, and dynamic adaptations to environmental challenges. Investigating these further measures could provide deeper insights into mobility decline and resilience in aging populations, ultimately leading to more tailored interventions to maintain functional independence.

In summary, our findings highlight the value of using validated DMOs to assess real-world walking in older adults, providing a reference that could guide interventions and improve quality of life. Finally, this population-based study can be used as a reference for studies involving specific pathologies to highlight differences between patterns of community-dwelling older adults and pathological patterns.

## Methods

### Study protocol

The study is based on data from the 4th and 5th follow-ups of the InCHIANTI study (clinical trial: NCT01331512). Two hundred community-dwelling older adults (79.5 ± 6.7 years), 49% female (Table 5, Supplementary Table 1), were monitored using a smartphone embedded with a tri-axial accelerometer and gyroscope (100 Hz sampling frequency), worn on the lower back in a belt. Participants received a dedicated smartphone (Samsung Galaxy SII, SIII) and information on how to use and wear it (using a belt). They were instructed to wear it after dressing, from morning to night, during their usual daily activities, for a one-week monitoring period. Clinical information was gathered during the baseline assessment at the clinic. The Ethical Committee of the Italian National Institute of Research and Care of Aging^18^ approved the InCHIANTI study protocol. The protocols complied with the Declaration of Helsinki. All participants received a detailed description of the study purpose and procedures and gave their written informed consent.

**Table 5:** Participants’ characteristics

	Study population	
Number of participants, *n*	200	
Age (years), mean (sd)	79.5 (6.8)	
Sex (F/M), *n* (% females)	98/102 (49%)	
Weight (kg), mean (sd)	71.3 (13.4)	
Height (m), mean (sd)	1.6 (0.1)	
BMI, mean (sd)	27.6 (4.0)	
Using a walking aid, *n* (%)	19 (9.5%)	
Education, *n* (%)	None: 7 (3.5%) Elementary: 106 (53.0%) Secondary: 38 (19.0%)	High School: 28 (14.0%) Professional school: 13 (6.5%) University: 8 (4.0%)
Clinical Gait Speed 4 m (m/s), mean (sd)	Usual pace: 1.1 (0.2) Fast pace: 1.4 (0.3)	
Hand grip (kg), mean (sd)	28.1 (9.1)	

### Data processing

The sensor data were first standardized according to the protocol outlined by Palmerini et al.^12^. Identification of walking bouts and extraction of gait features (initial contact, cadence, stride length) for these bouts were carried out using the validated Mobilise-D processing pipeline. The validation of this processing pipeline, based on a minimum 95% CI ICC threshold for performance metrics (i.e., sensitivity, positive predictive value, accuracy, etc.) of 0.7 and a relative error of less than 20%, is described in refs. ^13,15^. This enabled the calculation of 6 digital mobility outcomes (DMOs) for each walking bout (WBs): duration, number of strides, cadence, walking speed, step/stride length, and step/stride duration. A local version of this processing pipeline based in Matlab R2022b ran the algorithms in the analytical sequence described above.

Twenty-four walking activity and gait parameters were derived from the identified WBs (see Table 6)^34,38^. Measurements derived from the extracted gait DMOs were aggregated daily and then averaged over the week, resulting in a single value per week for each DMO and participant^34^. We removed days that did not meet the predefined minimum wear time required for a valid day (>12 h of waking time), and we considered a valid week (≥3 days, no weekday/weekend restrictions), as previously described in refs. ^38,39^. For this analysis, wearing time information was calculated based on a validated non-wear algorithm using the raw acceleration signals^40^.

**Table 6:** Digital mobility outcomes’ definition

Parameter	Unit	Daily calculation	Weekly calculation
Amount			
Walking duration	h/day	Daily sum of WBs duration using all WBs	Weekly mean of daily sum of WBs duration using all WBs
Number of steps	#/day	Sum of steps using all WBs	Weekly mean of daily sum of steps using all WBs
Pattern			
Number of WBs	#/day	Daily sum of all WBs	Weekly mean of daily sum of all WBs
Number of WBs > 10 s	#/day	Daily sum of number of WBs longer than 10 s	Weekly mean of daily sum of number of WBs longer than 10 s
Number of WBs > 30 s	#/day	Daily sum of number of WBs longer than 30 s	Weekly mean of daily sum of number of WBs longer than 30 s
Number of WBs > 60 s	#/day	Daily sum of number of WBs longer than 60 s	Weekly mean of daily sum of number of WBs longer than 60 s
WBs duration	s	Daily median of WBs duration using all WB	Weekly mean of daily median of WBs duration using all WBs
P90 WBs duration*	s	Daily P90 of WBs duration using all WB	Weekly mean of daily P90 of WBs duration using all WBs
WBs duration bout to bout variability	-	Daily COV of WBs duration using all WBs	Weekly mean of daily COV of WBs duration using all WBs
Pace			
Walking speed in shorter (10–30 s) WBs	m/s	Daily mean of walking speed using WBs between 10 s and 30 s	Weekly mean of daily mean walking speed using WBs between 10 s and 30 s
Walking speed in longer (>30 s) WBs	m/s	Daily mean of walking speed using WBs longer than 30 s	Weekly mean of daily mean walking speed using WBs longer than 30 s
P90 walking speed in WBs > 10 s*	m/s	Daily P90 of walking speed using WBs longer than 10 s	Weekly mean of daily P90 of walking speed using WBs longer than 10 s
P90 walking speed in longer (>30 s) WBs*	m/s	Daily P90 of walking speed using WBs longer than 30 s	Weekly mean of daily P90 of walking speed using WBs longer than 30 s
Stride length in shorter (10–30 s) WBs	cm	Daily mean of stride length using WBs between 10 s and 30 s	Weekly mean of daily mean of stride length using WBs between 10 s and 30 s
Stride length in longer (>30 s) WBs	cm	Daily mean of stride length using WBs longer than 30 s	Weekly mean of daily mean of stride length using WBs longer than 30 s
Rhythm			
Cadence in all WBs	steps/min	Daily mean of cadence using all WBs	Weekly mean of daily mean of cadence using all WBs
Cadence in longer (>30 s) WBs	steps/min	Daily mean of cadence using WBs longer than 30 s	Weekly mean of daily mean of cadence using WBs longer than 30 s
P90 cadence in longer (>30 s) WB*	steps/min	Daily P90 using WBs longer than 30 s	Weekly mean of daily P90 using WBs longer than 30 s
Stride duration in all WBs	s	Daily mean of stride duration using all WBs	Weekly mean of daily mean of stride duration using all WBs
Stride duration in longer (>30 s) WBs	s	Daily mean of stride duration using WBs longer than 30 s	Weekly mean of daily mean of stride duration using WBs longer than 30 s
Bout-to-bout variability			
Walking speed bout to bout variability in longer (>30 s) WBs	-	Daily COV of walking speed using WBs longer than 30 s	Weekly mean of daily COV of walking speed using WBs longer than 30 s
Stride length bout to bout variability in longer (>30 s) WBs	-	Daily COV of stride length using WBs longer than 30 s	Weekly mean of daily COV of stride length using WBs longer than 30 s
Cadence bout to bout variability	-	Daily COV using all WBs	Weekly mean of daily COV using all WBs
Stride duration bout to bout variability	-	Daily COV of stride duration using all WBs	Weekly mean of daily COV of stride duration using all WBs

^*^The 90th percentile (P90) is also referred to as ‘Maximum’ in refs. ^34,38^.

### Statistical analyses

All digital mobility outcomes (DMOs) were examined for normality using the Shapiro-Wilk test, with further inspection through histograms and skewness coefficients. DMOs that were confirmed as non-normal based on an absolute value of skewness coefficient greater than two were log-transformed^41^. Descriptive statistics, including means, standard deviations, medians, and interquartile ranges, were computed for each DMO and visualized as distributions.

Participants were divided into three age groups (65–74, 75–84, and 85–94 years) to assess subgroup differences. Sex differences in each DMO were evaluated using Analysis of Covariance (ANCOVA), adjusting for height and weight as covariates. To further assess potential differences in the distributions of height and BMI by sex, the Kolmogorov-Smirnov test was applied. Further analyses categorized participants within each sex as ‘short’ or ‘tall’ based on the median height for males and females, respectively, to assess the influence of height on DMOs. ANCOVA was again applied within these height categories to examine DMO differences while adjusting for age and weight, isolating the effect of height within each sex.

To explore relationships among DMOs and reduce dimensionality, we examined pairwise correlations and conducted network analysis and factor analysis. For factor analysis, factor loadings were assessed to assign DMOs to factors, with each DMO assigned to the factor with the highest loading, provided the loading exceeded 0.4. Where DMOs loaded onto multiple factors, the factor with the strongest loading was selected. Heatmaps were generated for each age group, allowing visualization of DMO interrelations and highlighting potential clusters.

To further investigate potential relationships between DMOs and age, we employed generalized additive models (GAMs), fitting models separately for males and females to capture sex-specific trends. Each GAM included a smooth term for age, capturing non-linear age effects and terms for height and BMI to control for these covariates. For each DMO, we reported the explained deviance (pseudo-${R}^{2}$) as an indicator of model fit and the *p*-value associated with the age term to assess its significance. DMOs were visualized in partial dependence plots with 95% confidence intervals to illustrate these non-linear age trends.

Finally, all *p*-values were adjusted for multiple comparisons using the false discovery rate (FDR) method by Benjamini-Hochberg to control for type I errors across multiple tests. Statistical analyses were conducted using Python 3.8, with key libraries including statsmodels, pyGAM, and seaborn for data visualization and model fitting.

## Supplementary information

Supplementary Material

## Data Availability

Data from the InCHIANTI study is available upon reasonable request, subject to the approval of the data controllers at https://www.nia.nih.gov/inchianti-study. Algorithms to identify walking events are available from the official Mobilise-D repository at https://github.com/mobilise-d/. Code for statistical analysis is available upon reasonable request.

## Code availability

Algorithms to identify walking events are available from the official Mobilise-D repository at https://github.com/mobilise-d/. Code for statistical analysis is available upon reasonable request.
