# Supplementary material for: Walking into aging: real-world mobility patterns and digital benchmarks from the InCHIANTI Study
Source: NPJ Aging. 2025 Jul 5;11(1):60. doi: 10.1038/s41514-025-00245-w (PMC12227531; doi:10.1038/s41514-025-00245-w)
Supplement: Supplementary file 1 — Supplementary Material [file 41514_2025_245_MOESM1_ESM.pdf]

Walking into Aging: Real-World Mobility Patterns and Digital Benchmarks from the InChianti Study

Jose Albites-Sanabria[1], Pierpaolo Palumbo[1], Stefania Bandinelli[2], Ilaria D'Ascanio[1], Sabato Mellone[1,3], Anisoara Paraschiv-Ionescu[4], Arne Küderle[5], Andrea Cereatti[6], Silvia Del Din[7,8], Felix Kluge[9], Eran Gazit[10], Carl-Philipp Jansen[11,12], Laura Delgado-Ortiz[13,14,15], Judith Garcia-Aymerich[13,14,15], Brian Caulfield[16], Lynn Rochester[7,8], Jochen Klenk[10,17,18], Luigi Ferrucci[19], Clemens Becker[10,11], Lorenzo Chiari[1,3], and Luca Palmerini[1]

Supplementary Material

Table 1. Health Conditions

Health Conditions: n (%)	Blindness: 1 (0.5%)<br>Cancer: 16 (8.0%)<br>Congestive Heart Failure: 3 (1.5%)<br>Diabetes: 35 (17.5%)<br>Hearing aid: 19 (9.5%)<br>Hip fracture: 2 (1.0%)<br>Osteoporosis: 41 (20.5%)<br>Paresis: 2 (1.0%)<br>Users with lower limb prostheses: 4 (2.0%)<br>Stroke: 11 (5.5%)

Table 2. Generative additive models generated for each DMO adjusted for height and BMI

DMO Label	Female (n=98)		Male (n=102)	
	p-value	pseudo-R^2	p-value	pseudo-R^2
Walking duration [h/day]	**0.004**	0.41	**<0.001**	0.45
Number of steps [steps/day]	**0.004**	0.43	**<0.001**	0.45
Number of WB [WB/day]	**0.004**	0.39	**0.001**	0.42
Number of WB > 10s [WB/day]	**0.004**	0.40	**<0.001**	0.44
Number of WB > 30s [WB/day]	**0.008**	0.39	**<0.001**	0.47
Number of WB > 60s [WB/day]	0.214	0.28	**0.011**	0.36
WB duration [s]	0.383	0.23	0.184	0.19
P90 WB duration [s]	0.116	0.31	0.623	0.12
WB duration bout to bout variability [-]	0.635	0.22	0.149	0.19
Walking speed in shorter (10-30s) WB [m/s]	**0.038**	0.44	0.062	0.39
Walking speed in longer (>30s) WB [m/s]	**0.006**	0.47	**0.001**	0.46
P90 walking speed in WB > 10 s [m/s]	**0.002**	0.54	**<0.001**	0.53
P90 walking speed in longer (>30s) WB [m/s]	**0.004**	0.47	**<0.001**	0.49
Stride length in shorter (10-30s) WB [cm]	0.076	0.46	**0.007**	0.44
Stride length in longer (>30s) WB [cm]	0.076	0.41	**<0.001**	0.52
Cadence in all WB [steps/min]	0.222	0.20	0.452	0.18
Cadence in longer (>30s) WB [steps/min]	0.084	0.35	**0.035**	0.31
P90 cadence in longer (>30s) WB [steps/min]	**0.011**	0.40	**0.035**	0.33

Stride duration in all WB [s]	0.278	0.19	0.062	0.26
Stride duration in longer (>30s) WB [s]	0.329	0.38	0.062	0.29
Walking speed bout to bout variability in longer (>30s) WBs [-]	0.139	0.31	**0.020**	0.33
Stride length bout to bout variability in longer (>30s) WBs [-]	0.220	0.27	0.309	0.25
Cadence bout to bout variability [-]	0.061	0.30	**<0.001**	0.40
Stride duration bout to bout variability [-]	0.435	0.22	0.258	0.17

Supplementary Table 2 presents the results of the Generalized Additive Models (GAMs) fitted to assess the non-linear relationship between each DMO and age, stratified by sex and adjusted for height and BMI.

Among the DMOs, walking duration, number of steps, and number of walking bouts (WB) per day showed strong associations with age in both females and males, with p-values below 0.01 and pseudo-R^2 values ranging from 0.39 to 0.45. For females, the number of WBs longer than 30 s showed a p-value of 0.008, while for males, the same DMO had a more pronounced association with a p-value of <0.001 and a pseudo-R^2 of 0.47, suggesting a stronger age-related trend in males. Walking speed in both shorter (10-30 s) and longer (>30 s) walking bouts showed significant non-linear trends with age, with higher pseudo-R^2 values for males (up to 0.46). P90 walking speed within longer bouts was similarly associated with age, with p-values below 0.01 and pseudo-R^2 values up to 0.54 for females and 0.53 for males. On the other hand, in males, stride length during longer walking bouts had a significant age effect (p<0.001) with a pseudo-R^2 of 0.52. Cadence-related DMOs showed mixed results, with cadence in longer bouts showing significant differences in both sexes, while overall cadence did not show a significant relationship in either group. Finally, variability measures demonstrated less consistent associations. Among these, walking speed variability in longer bouts was significantly different in males (p=0.020, pseudo-R^2 = 0.33), whereas other variability metrics showed weaker or non-significant differences in both groups.

Supplementary Figures

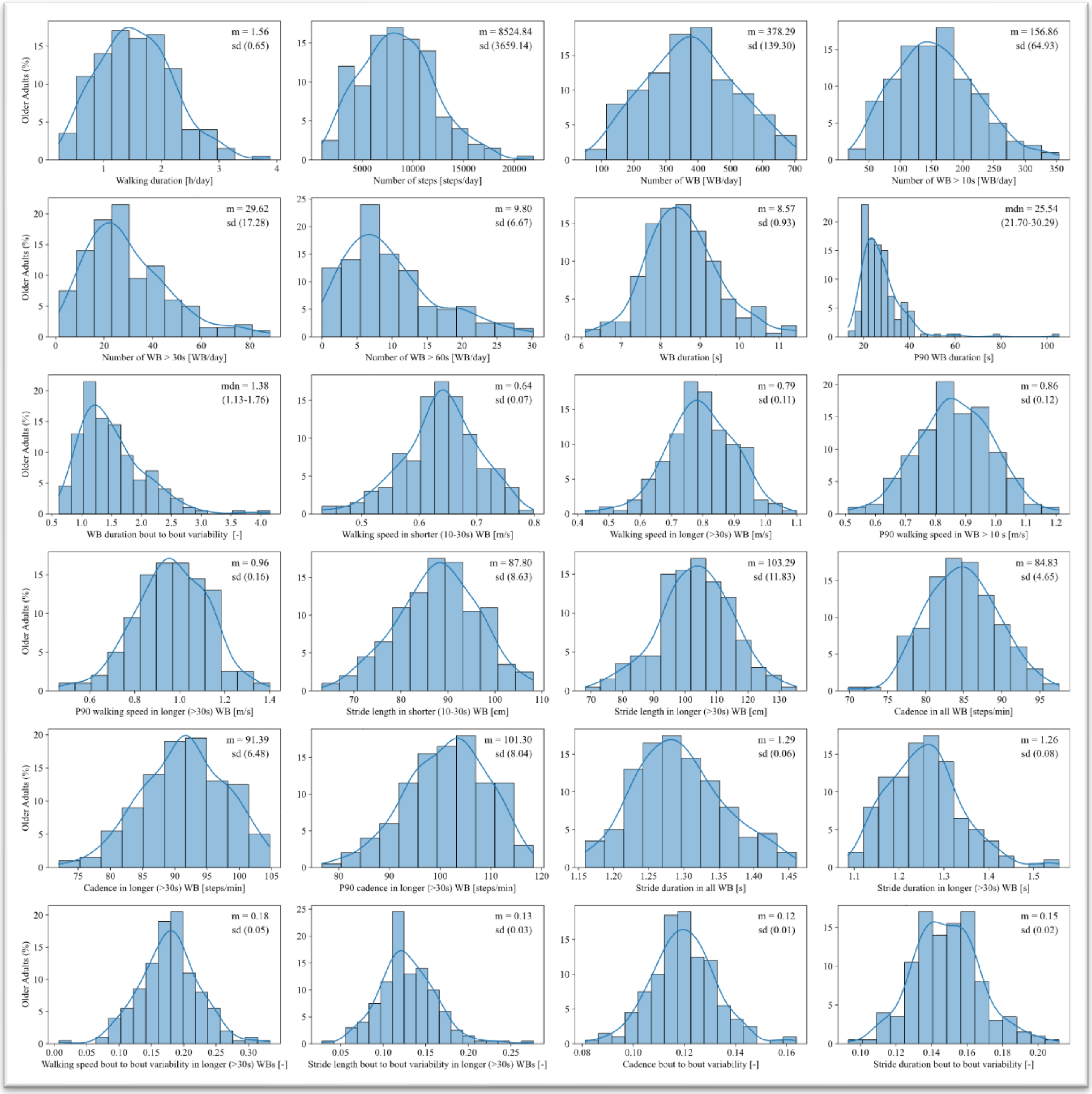

Figure 1. Digital mobility outcomes' distribution

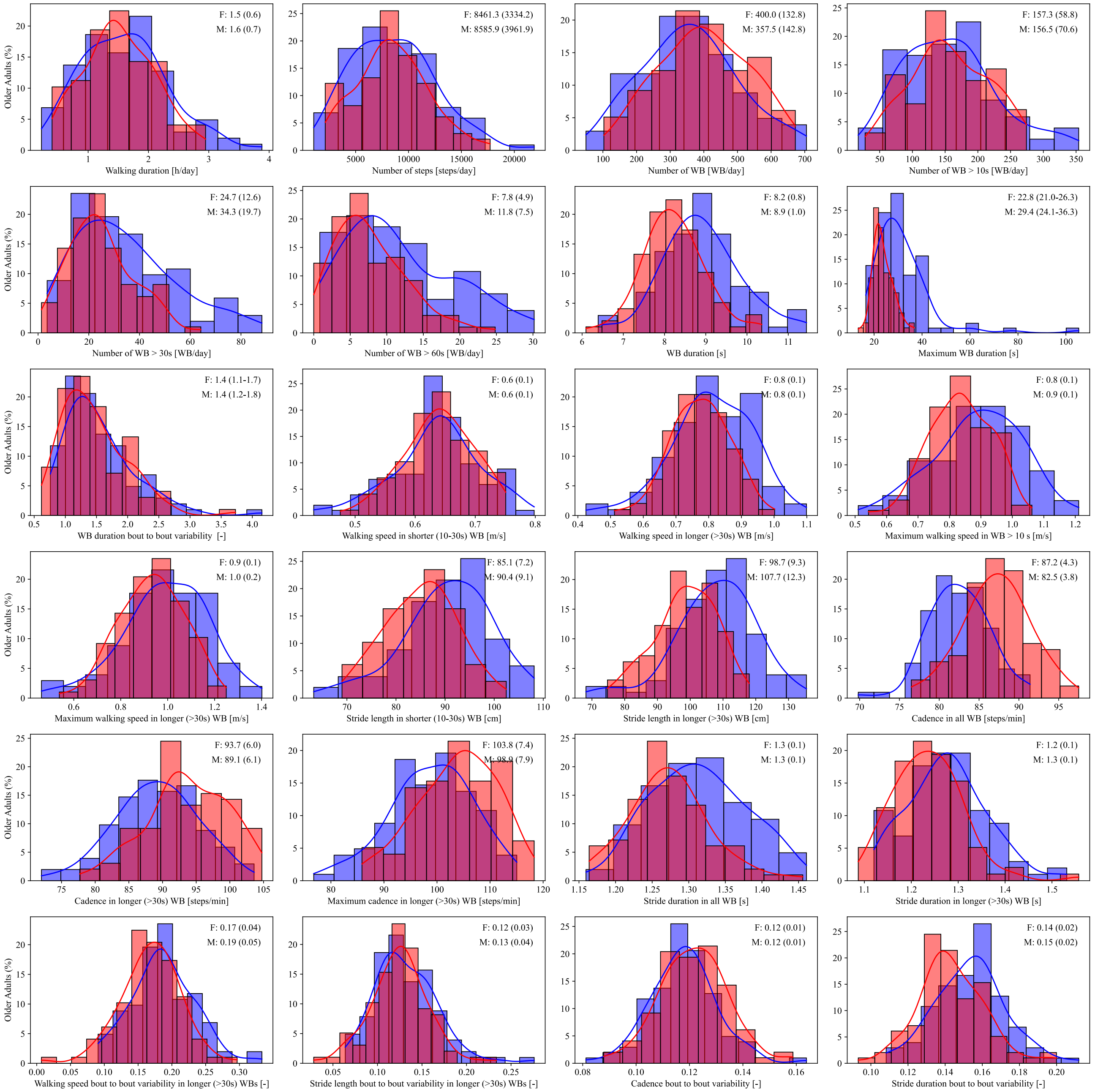

Figure 2. Digital mobility outcomes' distribution, females (F) in red, males (M) in blue

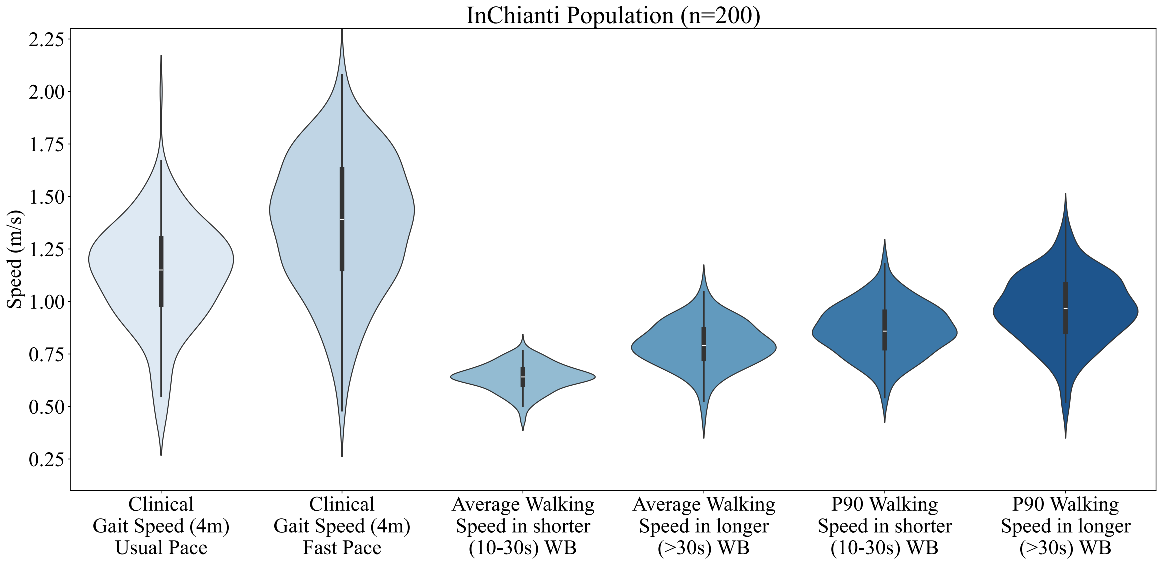

Figure 3. Box and Violin plots for clinical and real-world walking speeds for all participants
